# Case Report: Superior mesenteric artery vasculitis causing aneurysm following COVID-19 infection

**DOI:** 10.3389/fsurg.2024.1394638

**Published:** 2024-08-21

**Authors:** Minju Kim, Jeong Hee Han, Jung Bum Choi, Byoung Chul Lee, Hyuk Jae Jung

**Affiliations:** Department of Surgery, Biomedical Research Institute, Pusan National University Hospital, Pusan National University School of Medicine, Busan, Republic of Korea

**Keywords:** superior mesenteric artery, aneurysm, arteritis, COVID-19, steroid

## Abstract

**Objective:**

Arteritis refers to all infectious and non-infectious conditions that lead to inflammation of the arterial wall. However, little is known about its presence in patients with coronavirus disease 2019 (COVID-19). Most patients improved with steroids along with conservative treatments in a few studies. We report our experience with superior mesenteric artery (SMA) arteritis causing an aneurysm following COVID-19 infection.

**Case presentation:**

A 66-year-old female patient who was infected with COVID-19 1 month prior presented with abdominal pain. A computed tomography scan revealed proximal SMA arteritis. Although preliminary antibacterial treatment was initiated, the follow-up CT revealed an aggressive and fast-growing 5.7-cm SMA aneurysm. Subsequently, an open interposition bypass of the SMA aneurysm was performed successfully. As the specimens retrieved during surgery showed no bacterial colonization in the tissue or blood cultures, the patient was discharged without complications.

**Conclusions:**

The mechanism of arteritis in patients with COVID-19 has not been elucidated. In the absence of evidence of bacterial infection in arteritis, it is necessary to consider the possibility of viral infection caused by COVID-19 during the COVID-19 pandemic era and start with high-dose steroid therapy promptly.

## 1 Introduction

Arteritis refers to all infectious and non-infectious conditions that lead to inflammation of the arterial wall (1). Various etiologies dictate the variable presentations and outcomes in patients with arteritis. The clinical presentation varies across a spectrum of symptoms and clinical signs, and arteritis in major vessels can cause ischemia or infarction in the distal tissue. Pyogenic arteritis, caused by *Salmonella* and *Staphylococcus aureus*, is the most common form of infectious arteritis, whereas non-infectious arteritis includes Takayasu arteritis and giant cell arteritis (2). Since arteritis may be asymptomatic and/or manifest with atypical symptoms, its prevalence related to a specific disease cannot be extrapolated from sparse epidemiological studies (1). Recently, cases of arteritis have been reported after coronavirus disease 2019 (COVID-19) infection, and most of them improved with steroids and conservative treatments. In rare cases, it progresses to a serious condition, and we report our experience with superior mesenteric artery (SMA) arteritis causing an aneurysm following COVID-19 infection.

## 2 Case description

**Figure 1:**
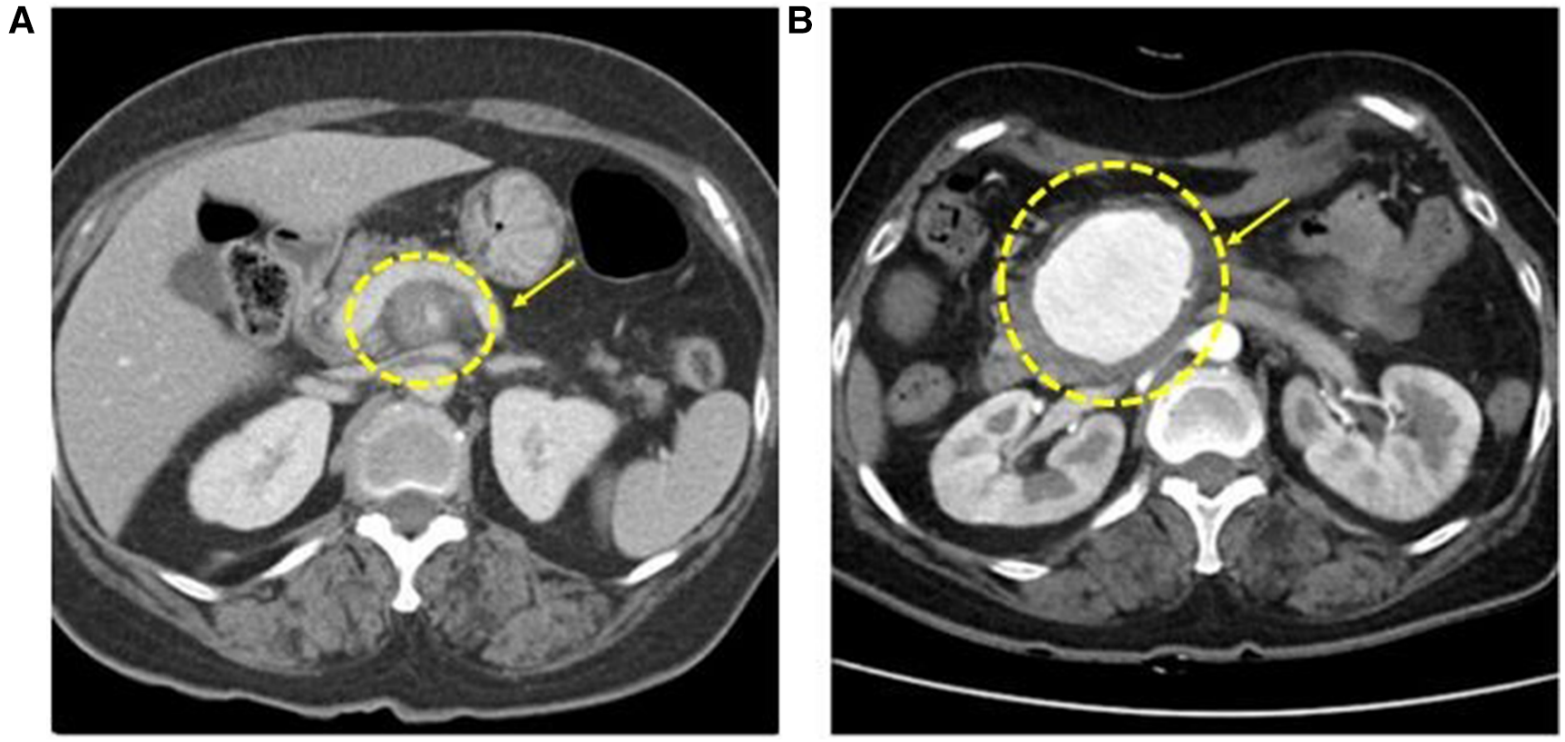
Initial CT angiography shows superior mesenteric artery (SMA) inflammation surrounding fat stranding around the proximal SMA **(A)**. After 25 days of follow-up, CT angiography presents a 5.7 cm aneurysm on SMA **(B)**.

A 66-year-old female patient with a history of hypertension and dyslipidemia presented to the emergency department with suspected arteritis. According to previous hospital records, the patient had a history of COVID-19 infection 6 weeks before visiting the other hospital and had discomfort in the upper abdomen 4 weeks before visiting the hospital. On the day of the visit, the abdominal pain suddenly intensified, and an abdominal computed tomography (CT) scan was performed, which confirmed inflammation in the proximal SMA (Figure 1A).

Her initial vital signs were within normal ranges. Physical examination revealed mild epigastric tenderness without rebound tenderness, visible distension, or other abnormalities. Laboratory findings showed non-specific leukocytosis with an elevated C-reactive protein (CRP) level of 8.09 mg/dl and an electrolyte sedimentation rate of 120 mm/h. The rheumatic factor test performed together showed non-specific findings, including C3 of 188.0 mg/dl, C4 of 33.4 mg/dl, FANA of <1:80, MPO Ab negative, and PR-3 Ab negative. As the rheumatic test was negative and the initial impression was pyogenic arteritis, vancomycin and ceftriaxone were the first administered empirical antibiotics, and blood cultures were conducted every 3 days in both the peripheral and central veins.

**Figure 2:**
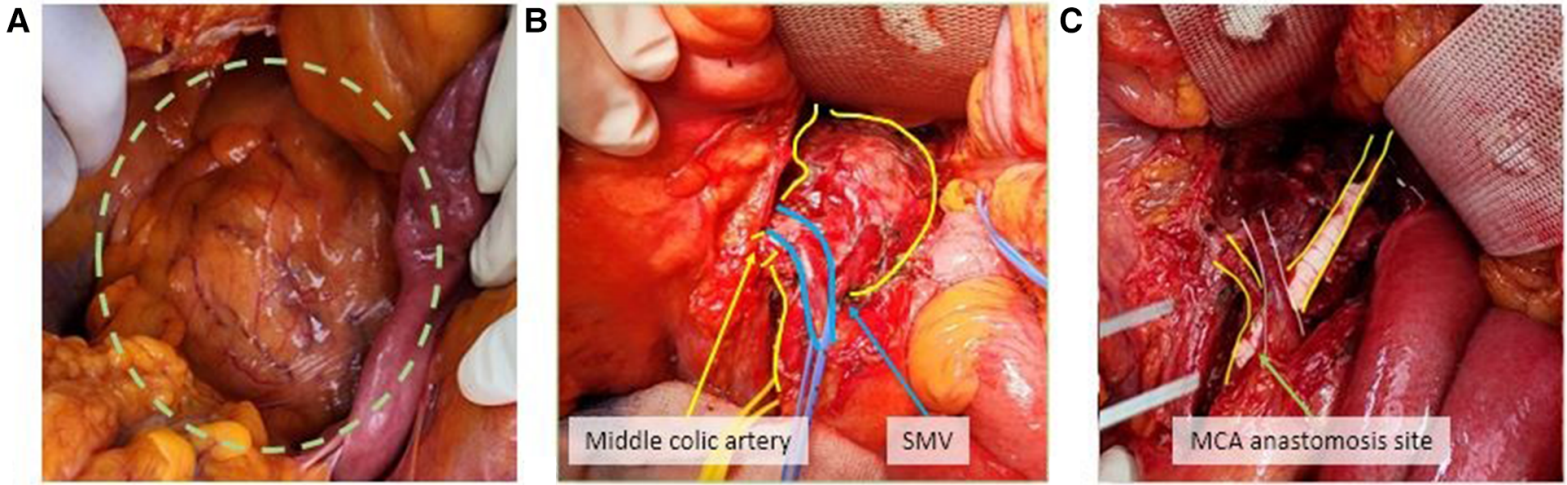
A 5.7 cm superior mesenteric aneurysm was identified in operation **(A)**. The superior mesenteric vein passed over the aneurysm sac and the branch of the mid-colic artery was confirmed **(B)**. SMA interposition bypass using a PTFE graft with mid-colic artery bypass was done **(C)**.

**Figure 3:**
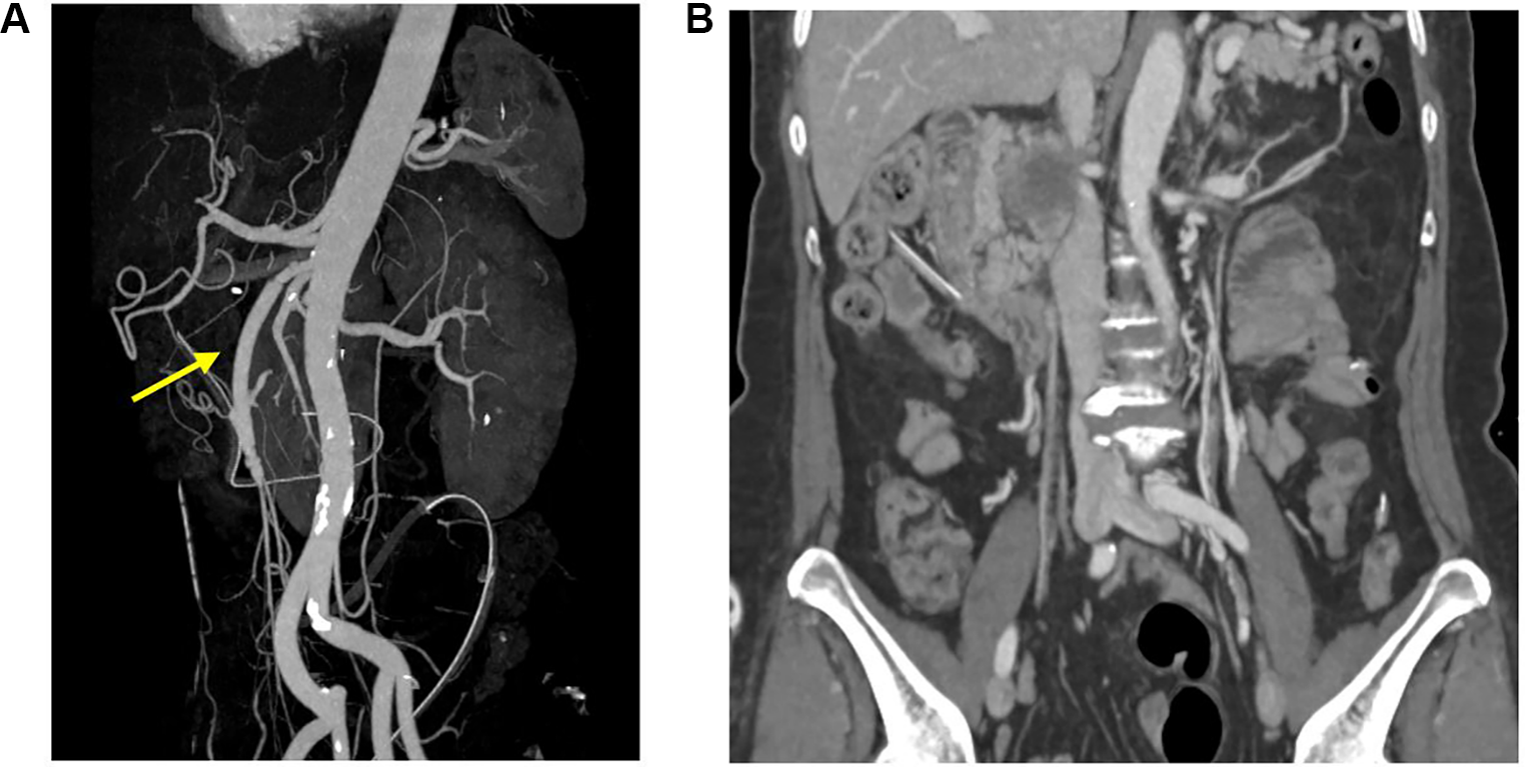
Post operation follow-up CT angiography showed recanalization of superior mesenteric artery. (arrow) **(A)**, and blood flow was intact without ischemic changes in the bowel **(B)**.

After 1 week, follow-up CT angiography showed increased inflammatory lesions and aneurysmal dilatation in the proximal SMA. Despite the administration of antibiotics, the inflammatory lesions progressed rapidly, and causes other than bacteria, such as *Salmonella* or *S. aureus*, were considered. Since none of the blood culture tests confirmed bacterial colonies and there was no infection source other than COVID-19 infection history, vasculitis caused by viral infection was suspected, and steroid therapy was initiated. Methylprednisolone was administered at a dose of 1 mg/kg, after which the CRP level decreased. The steroid treatment lasted for 25 days, after which CT angiography was performed for follow-up observation. CT angiography revealed a 5.7 cm pseudoaneurysm on the proximal SMA, but the distal flow was intact (Figure 1B). Surgical resection and bypass of the aneurysm were planned. The area around the SMA aneurysm was gently dissected, and after moving from the distal to the proximal SMA, an aneurysmectomy was performed. Inflammatory tissue was observed in the area around the aneurysm, and a culture test was performed on the peri-aneurysmal tissue and thrombus. Using a 6 mm × 50 cm PTFE graft, a bypass was performed from the SMA orifice as end-to-end anastomosis, and mid-colic artery and graft were performed end-to-side anastomosis (Figure 2). After 7 days, the follow-up CT angiography showed that blood flow was intact without ischemic changes in the bowel (Figure 3). Specimens obtained during surgery showed no bacterial colonization in the tissue or blood culture. Postoperative care was completed, and the patient was discharged. The above time line is shown in Table 1.

**Table 1:** Timeline on the episode.

23/01/19	COVID-19(+) comfirmed	** **
23/03/19	Epigastir area tenderness CT evaluation on ER	r/o arteritis on proximal SMA
	Administration	Serial blood culture every 3 days Empirical antibiotic- vancomycin and ceftriaxone start
23/03/28	Follow-up CT angiography	Increased inflammation →Methyprednisolon 1 mg/kg start
23/04/18	Follow-up CT angiography	5.7 cm sized pseudoaneurysm on proximal SMA →Surgical resection and bypass done
23/04/24	Follow-up CT angiography	Blood flow intact without ischemic changes in the bowel
23/04/28	Discharge	** **

## 3 Discussion

Arteritis has various etiological causes. Primary large vessel vasculitides, giant cell arteritis, and Takayasu arteritis are the most common non-infectious causes, and in infectious arteritis, *Salmonella* spp. is the most commonly isolated pathogen, followed by *Staphylococcus and Streptococcus* spp. (3). Coexisting bacteremia is found in over 50% of cases, with evidence of isolated infection elsewhere, most commonly endocarditis (4). Antibiotic therapy combined with complete surgical excision of the infected artery is the best treatment for bacterial infectious arteritis (5).

Virus-related vasculitis with aortic involvement is rare, but an association between inflammatory vascular diseases and viral infections has been described in patients with hepatitis B and C (6, 7). A causal relationship has been firmly established in a few instances of vasculitis, such as hepatitis B virus-associated polyarteritis nodosa, and hepatitis C virus with cryoglobulinemic vasculitis. Although it is rare, there have been reports of HCV infection causing aortitis. In the case of varicella-zoster virus infection, it is reported that extracranial vasculopathy could produce transient ischemic attack, aneurysm, sinus thrombosis, and giant cell arteritis, as well as granulomatous aortitis (8–10). Treatments of virus-associated vasculitides mainly rely on antiviral agents. The indications for corticosteroids and/or other immunosuppressants are limited to virus-associated vasculitides, and their use should be short-term and considered as adjuvant therapy for refractory diseases and their most severe forms (6).

Since laboratory findings did not identify a non-infectious cause such as ANCA positive, this case was initially suspected to be bacterial infectious arteritis, and antibiotics were administered as the first treatment. Despite the use of antibiotics, the inflammatory lesion progressed rapidly on follow-up CT angiography. Therefore, other causes for arteritis had to be considered.

Moreover, blood culture tests did not confirm bacterial colonies, and there was no medical history other than the recent COVID-19 infection. Therefore, after excluding the other causes, COVID-19 infection was suspected as the only cause of arteritis. After establishing the history of COVID-19 infection, no respiratory infection symptoms such as fever, cough, phlegm, or events after the quarantine period were detected. Consequently, viral infection was overlooked during the initial evaluation as the cause of arteritis when the patient reported to us. Finally, despite the rare incidence of virus-related vasculitis, this case was suspected to be viral infectious vasculitis caused by COVID-19, and steroid treatment was initiated. In confirming an aneurysmal change on follow-up CT angiography for the first time, steroid treatment was administered, and the patient's clinical symptoms improved, with reduced CRP levels. However, due to the weakening of the arterial wall caused by the inflammatory change, an aggressive and fast-growing 5.7 cm SMA aneurysm was observed on a CT scan after 1 month. An infected aneurysm can rapidly develop or enlarge and subsequently undergo free rupture owing to sustained systemic arterial pressure (11). Therefore, surgical treatment was performed considering the risk of rupture.

According to previous reports, high-dose steroids are important for managing arteritis in COVID-19 (12). However, the appropriate timing for suitable treatment was most likely missed because the time to apply steroids was delayed owing to the exclusion of other infectious causes in this case.

Although the mechanism of arteritis in COVID-19 has not been fully elucidated, some studies showed that COVID-19 can directly infect endothelial cells, causing endothelial inflammation (13). Alternatively, Zou et al. (14) speculated that the cytokine storm observed in some patients with COVID-19 infection causes endothelial cell dysfunction and inflammation in cases with elevated interleukin-6. Other studies have proposed that inflammation can be mediated by an immune response rather than a direct consequence of the virus (12). Considering the clinical aspects of this case, it is highly likely that inflammation was caused by direct infection of endothelial cells.

There are a handful of cases wherein arteritis progresses to an aneurysm, and surgical treatment is rarely required. Arteritis affecting the aorta or major arteries can lead to life-threatening complications if proper treatment is not administered promptly. Herein, we discuss a patient suffering from an arteritis-induced aneurysm necessitating surgery. This case demonstrates the effectiveness of a quick diagnosis of the etiological cause for optimum treatment. As arteritis is associated with several predisposing factors, it is necessary to consider viral infection in a patient with a history of COVID-19 if the cause of arteritis is unknown during this COVID-19 pandemic era.

## Data Availability

The original contributions presented in the study are included in the article/Supplementary Material, further inquiries can be directed to the corresponding author.
